# The Predation Game: Does dividing attention affect patterns of human foraging?

**DOI:** 10.1186/s41235-021-00299-w

**Published:** 2021-05-06

**Authors:** Ian M. Thornton, Jérôme Tagu, Sunčica Zdravković, Árni Kristjánsson

**Affiliations:** 1 Department of Cognitive Science, Faculty of Media and Knowledge Sciences, University of Malta, Msida, Malta; 2 Faculty of Psychology, School of Health Sciences, University of Iceland, Oddi v. Sturlugötu, 101, Reykjavik, Iceland; 3 EA 4139 Laboratory of Psychology, University of Bordeaux, Bordeaux, France; 4 Department of Psychology, Faculty of Philosophy, University of Novi Sad, Novi Sad, Serbia; 5 Laboratory for Experimental Psychology, University of Belgrade, Belgrade, Serbia; 6 School of Psychology, National Research University, Higher School of Economics, Moscow, Russian Federation

**Keywords:** Foraging, Predation, Visual search, Divided attention, Multiple target search, Dual-task, Multiple-object tracking

## Abstract

Attention is known to play an important role in shaping the behaviour of both human and animal foragers. Here, in three experiments, we built on previous interactive tasks to create an online foraging game for studying divided attention in human participants exposed to the (simulated) risk of predation. Participants used a “sheep” icon to collect items from different target categories randomly distributed across the display. Each trial also contained “wolf” objects, whose movement was inspired by classic studies of multiple object tracking. When participants needed to physically avoid the wolves, foraging patterns changed, with an increased tendency to switch between target categories and a decreased ability to prioritise high reward targets, relative to participants who could safely ignore them. However, when the wolves became dangerous by periodically changing form (briefly having big eyes) instead of by approaching the sheep, foraging patterns were unaffected. Spatial disruption caused by the need to rapidly shift position—rather the cost of reallocating attention—therefore appears to influence foraging in this context. These results thus confirm that participants can efficiently alternate between target selection and tracking moving objects, replicating earlier single-target search findings. Future studies may need to increase the perceived risk or potential costs associated with simulated danger, in order to elicit the extended run behaviour predicted by animal models of foraging, but absent in the current data.

## Introduction

Traditional visual search—involving a single target and a variable set-size of distractors—has taught us much about the cognitive processes we use to successfully locate items of interest in the world around us (Treisman & Gelade, 1980; Wolfe & Horowitz, 2017). Extending the classic single-target paradigm, a number of groups have also examined search behaviour in tasks where multiple targets must be located on a given trial (see Kristjánsson et al., 2019; Thornton et al., 2020 for recent discussion). Much of this work stems from the observation that real-life activities—such as finding the correct change, shopping in a store, assembling a new piece of furniture—often involve a series of identification and selection events. In our own work (e.g.,. Kristjánsson et al., 2014), we have taken inspiration directly from the selection behaviour of foraging animals. In particular, we have argued that common attentional constraints may account for the similar behavioural patterns seen in animal foraging and human multiple-target search scenarios (Kristjánsson et al., 2014).

Continuing this line of foraging research, the current paper addresses another important aspect of search in the real-world: the fact that we rarely have the luxury of being able to focus attention solely on target selection. For humans, we may be distracted by ongoing conversations, keeping an eye on the kids or simply responding to the ever-present mobile phone. For animals, one of the most frequent causes of distraction—and the one that serves as inspiration for the current work—is the risk of predation (e.g., Abrahams & Dill, 1989; Brown, 1988; Gilliam & Fraser, 1987; Kotler, 1984; Lima, 1998; Sih, 1982).

For many organisms, risk of predation can affect both the quantity and the quality of foraging episodes. For example, there may be a direct reduction in activity and/or a change to usual exploration patterns, in order to avoid predators. While effective, such changes can also have serious repercussions in terms of energy uptake, and can therefore affect survival rate and reproductive success (Abrahams & Dill, 1989; Houston et al., 1993; Kotler & Brown, 2017; Laundre et al., 2010; Lima, 1998; Lima & Dill, 1990). Of particular relevance to the current paper, when animals become aware of possible predators they may be forced to divert significant attentional resources away from the foraging task, making them less efficient at finding appropriate food sources, particularly when items are less available or less visible (Dukas, 2004; Kotler et al., 2004).

**Fig. 1:**
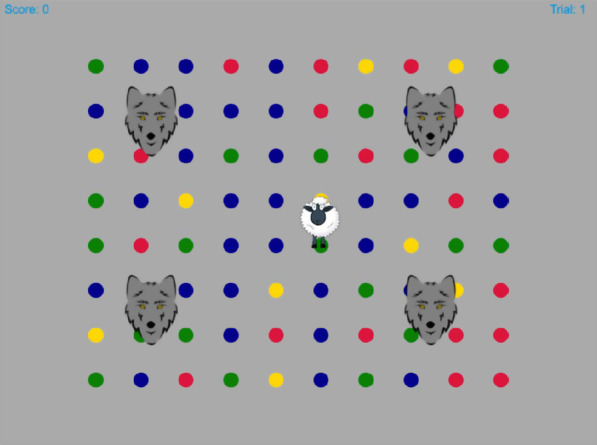
Screenshot of The Predation Game. See text for details

To explore divided attention in the context of human foraging, we created an online game that closely mirrored the task demands of our original studies (Kristjánsson et al., 2014; Thornton, 2019) while also simulating the risk of predation (Fig. 1). Participants were asked to cancel items from two target categories using a “sheep” icon, while either avoiding or ignoring (depending on condition) a pack of “wolf” objects that also roamed the screen. For participants in the “hunted” condition, contact with any wolf terminated the trial. For participants in the “distracted” condition, the wolves did not interact with the sheep and could only affect trial outcome by briefly masking target items. For both groups, mistakenly selecting a distractor item terminated the trial. While Fig. 1 provides a static snapshot of a typical trial, the game can be played directly online at https://maltacogsci.org/thePredationGame.

Our general question was whether the foraging patterns of human participants would vary as a function of whether the wolf objects were “dangerous” or simply distracting. In the remainder of this introduction, we first briefly review what is known about typical patterns of human foraging obtained with multiple-target search tasks. We then consider how search patterns might be expected to change under dual-task conditions, before providing specific predictions based on the demands in the current predation scenario.^1^

### Attention & human foraging

Attention is thought to play an important role in shaping the behaviour of both human (Bond, 1982; Kristjánsson et al., 2014; Wolfe et al., 2019) and non-human (Dawkins, 1971; Dukas & Ellner, 1993; Kamil & Bond, 2006; Tinbergen, 1960) foragers. When attentional load is low, foraging behaviour is often unconstrained. For example, an animal might move freely through the environment, selecting food items at random from all available sources (Dukas, 2004). Similarly, when time is unlimited and targets are easy to identify, human participants select at random from the available target categories, scanning through displays according to individual preference (Kristjánsson et al., 2014). However, when attentional demands increase—for example, because prey are no longer conspicuous or targets are defined by a conjunction of features—item selection becomes less random. Specifically, both human and animal foragers are then more likely to choose items from the same category giving rise to characteristic “runs” of selection, that are often clearly visible in the raw data (Bond, 1983; Dawkins, 1971; Kristjánsson et al., 2014).

In our previous work, we have used simple, game-like 2D (e.g., Jóhannesson et al., 2016, 2017; Kristjánsson et al., 2014, 2018; Thornton et al., 2019) and 3D (Kristjánsson et al., 2020a, b; Prpic et al., 2019) tasks to explore such foraging behaviour in humans. As noted above, these studies form part of a more general research trend exploring multiple-target visual search in humans. To-date, selection difficulty (Kristjánsson et al., 2014), selection modality (Jóhannesson et al., 2016; Tagu & Kristjánsson, 2020; Thornton et al., 2019), patch-leaving (Wolfe, 2013), time constraints (Kristjánsson et al., 2018; Thornton et al., 2020) and reward (Wolfe et al., 2018) have all been used to directly modulate multiple-target search behaviour in humans.

While the measures of interest vary from study to study, our own previous work has focused primarily on patterns of foraging runs. A “run” in this context simply refers to a sequence of selections from the same target category. Typically, when attentional demands increase, the tendency to switch between target categories decreases, leading to a reduction in the number of runs. The goal of the current study was to determine whether having to divert attention away from the primary task of detecting targets to monitor other aspects of the display would also lead to a change in run behaviour. To our knowledge, this is the first time that a divided attention paradigm has been employed in the context of multiple-target search or human foraging.

### Search under dual-task conditions

A great deal is known about the effects of dividing attention on standard visual attention paradigms. While current technology increasingly calls for such dual task performance—nowadays we go about our daily tasks phone in hand—it is also well-known that attentional capacity is limited and attending to one aspect of a given task can lead to detriments in performing another task. People have great trouble attending to two simultaneous streams of speech (Broadbent, 1958; Cherry, 1953; Moray, 1959) or two or more simultaneous streams of visual information (Kristjánsson & Nakayama, 2002; Reeves & Sperling, 1986). Multiple object tracking (MOT) tasks (Meyerhoff et al., 2017; Pylyshyn & Storm, 1988), where observers have to keep track of a number of items moving around on the screen, reveal that observers can only keep track of a limited number of items and this most likely reflects limited attentional capacity (Alvarez & Franconeri, 2007; Franconeri et al., 2008; Scholl, 2009).

In terms of visual search under dual-task conditions, many studies have focused on the influence that concurrent memory load has on performance (see Olivers et al., 2011 for review). Perhaps surpringly—given the central role that visual working memory (VWM) is thought to play in many theories of visual search, evidence for substantial dual-task costs is rather sparse. For example, Woodman et al. (2001) found no change in search efficiency—defined as an increase in reaction time as a function of set size—when search was carried out in isolation as compared to during the retention interval of an object memory task.

Drew et al (2016) extended these findings to the context of “hybrid search” (Schneider & Shiffrin, 1977; Wolfe, 2012), where targets change from trial to trial drawn from a memorised set held in long-term memory (LTM). In seven experiments they found little to no effect of working memory load on search efficiency, leading them to suggest that, at least during hybrid tasks, VWM may only serve to pass the current object of attention to later areas of “activated” LTM for further processing (Drew et al., 2016). While it does appear that loading spatial working memory—asking participants to remember the location of objects—can affect search efficiency (Oh & Kim, 2004; Woodman & Luck, 2004), the slope differences between single and dual-task search appear quite modest (20–30 ms/item) and do not generalise to hybrid search tasks, where there is no measurable effect (Drew et al., 2016; Expts. 4 & 5).

The study from the single-target search literature that most closely parallels our current work is that by Alvarez et al. (2005). In a series of experiments, they used an attention operating characteristic (AOC) methodology (Sperling & Dosher, 1986; Sperling & Melchner, 1978) to determine whether MOT and visual search draw continuously on the same attentitional resources. They found that while both tasks could be completed within the same trial quite successfully, there were small but measurable dual-task costs. Furthermore, the nature of these dual-task costs was most consistent with the idea that participants used executive control to rapidly switch the same resources back and forth between the two tasks. Thus, items can be tracked while searching, but only by temporarily withdrawing resources from the task of selecting targets. How might such task switching affect patterns of human foraging?

### Current task demands & predictions

The online task used in the current study involes a dual-task design where two challenging continuous attentional tasks are pitted against one another: (1) attentional selection of multiple targets among distractors (the foraging task) and (2) tracking/monitoring task involving mutliple objects (the wolves in our case) where collisions need to be avoided. There are clearly some important differences between this design and the study of Alvarez et al (2005).

First, each foraging trial is expected to last between 20 and 30 s, rather than 5 s in the single-target design, during which time 40 target selection events are required. The difficulty of selection was varied across blocks of trials, using either single-feature (colour) or conjunction (colour and shape) targets. Throughout this extended search period, “hunted” participants were required to monitor for the approach of four wolf objects, and to take avoiding action to prevent a collision. While the tracking component of this task is reduced compared to standard MOT—there is no requirement to continuously track the position of all four predator objects—the task is nonetheless demanding due to the need to actively avoid collisions (Thornton et al., 2014). Another group of participants (the “distracted” group) could, on the other hand ignore the wolves as they posed no threat to them.

Overall, then, based on previous dual-task studies in humans, and the expected behaviour of animals exposed to risk of predation (Dukas, 2004; Kotler et al., 2004), our main prediction was that participants who had to actively avoid collision with the predator objects would display patterns of foraging that reflected an increase in attentional load. Specifically, that they would switch less often between target categories, leading to fewer, longer runs than those patricipants who were not being hunted. In having to modify their search patterns to avoid the “dangerous” objects, we also expected the movement of hunted participants through the display to be less systematic and possibly slower overall than of those participants who could effectively ignore the wolves.

## Experiment 1: Hunting while hunted

In our first experiment 48 participants completed the foraging game online. We manipulated risk of predation between subjects, with 24 participants in the distraction condition, and 24 in the hunted condition. We also modulated task difficulty in a number of other ways. In separate blocks, target selection was based on either a single colour feature or on a conjunction of colour and shape, as in our previous work (Kristjánsson et al., 2014). Across trials, we also varied the velocity with which the wolf objects moved, to increase or decrease the risk they posed. Finally, for half of the participants in each predation group we varied the behaviour of the wolf objects. For those in the “pack” condition, all 4 predator objects moved with independent, linear trajectories, irrespective of the position of the sheep object. For those in the “lone” wolf condition, one of the 4 wolf objects always changed direction to follow the current position of the sheep object. The other 3 wolves moved with independent linear trajectories, as in the pack condition. Again, this manipulation was included to increase the potential risk posed by the predators.

### Methods

#### Participants

All 48 participants were recruited online from https://prolific.co. They were required to be fluent readers of English, within a specified age range (18–40 years) and to have not taken part in previous related studies. Demographically, they were located in different countries, with different native languages, variously employed or in full-time study, aged from 18 to 40 years (*M* = 27.1 years, SD = 5.7), and 21 were female. For their participation in the experiment they were paid a flat rate of £3.75, based on an estimated session time of 30 min.

#### Ethics & data protection

The research team were unaware of and had no access to the personal identity of the participants. In addition to the implied consent—given that participants were recruited through a voluntary, professional service—a full information sheet and consent form was presented prior to data collection. Participants were given the option of downloading these documents for later reference. They were required to confirm that they had read and understood the nature of the experiment and the data that would be collected and to explicitly confirm their informed consent for participation. These online procedures conform to the Ethics and Data Protection guidelines of the University of Malta.

#### Power analysis

The basic group size (*N* = 12) was determined prior to data collection and was chosen to directly match recent studies from our group where within-subject differences in run behaviour had been successfully measured (Thornton et al., 2019, 2020). To further verify that this sample size would provide sufficient power to detect the within-group feature/conjunction foraging patterns of interest, we conducted an a priori power analysis using the “Bias and Uncertainty Corrected Sample Size” (BUCSS) toolbox described by Anderson et al. (2017). BUCSS uses the reported *F* values and sample size from previous factorial studies—rather than derived estimates of effect size—to generate necessary sample sizes for planned studies. Here, we chose the previous study from our group (Thornton et al., 2020) that most closely matched the current within-group factorial design. Specifically, we chose a 2 (Target: feature/conjunction) × 5 (Foraging Tempo) repeated measures analysis of variance conducted on run length with a sample size of 11, focusing our a priori analysis on the main effect of Target, *F*(1,10) = 40.0, *p* < 0.001, MSE = 6.3, ${\eta }_{p}^{2}$= 0.8. We used this *F* value, along with the sample size and alpha parameters from Thornton et al. (2020) as input to the BUCSS *ss.power.wa* function. We chose custom settings of assumed alpha for the planned study = 0.05, level of assurance = 0.95, and desired power of 0.8. We specified the main within-subject factors from the current experiment—2 (Target) × 5 (Wolf Velocity)—and identified the main effect of Target as the effect of interest. This analysis yielded a minimum sample size of 11 participants, closely approximating our initial choice.

#### Online protocols

All data for the current study were collected online. Participants were directed to a dedicated URL on the https://maltacogsci.org domain and were taken through a series of webpages that provided instructions, obtained consent and ran the experimental trials. Anonymous data was transferred automatically on a trial-by-trial basis to a secure server for later download and processing. As participation was remote, we could not control the specific laptop/desktop machines that were used, nor the monitor hardware/settings. We did exclude the use of mobile devices, as this version of our foraging task was designed not to respond to touch-based technology. We have previously run the basic foraging task in a desktop environment (Thornton et al., 2019), and while we anticipated some consequences on overall patterns of run behaviour related to reduced response selection speed (Thornton et al., 2020), these would be constant across the current manipulations of interest.

#### Equipment

As the current study was run online, we could not control the precise display conditions or equipment used. The online task was custom written in JavaScript so that it would run via web browsers opened on any laptop or desktop machine. Several recent review papers have indicated that the display and response timing of native JavaScript is capable of producing data that is comparable to lab-based testing (e.g., Bridges et al., 2020; Miller et al., 2018; Pronk et al., 2019). The code ensured that browsers were switched to full-screen mode, so that only the foraging display appeared centered on the screen. Checks within the code identified the physical frame rate of the display and capped the effective update rate to 60 Hz. To minimize possible mouse versus trackpad differences in response times, participants were allowed to move the cursor with either, but observers were required to press the spacebar to register a response. We have used this technique previously to equate response demands across input modalities (Thornton et al., 2019).

#### Foraging stimuli

Figure 1 shows the initial moment of a typical trial. Stimuli appeared on a grey canvas region (800 × 600 pixels) that was always centred on an otherwise blank, full screen. Participant used their regular mouse/trackpad to control the position of the cursor, that was visualised as a sheep (64 × 80 pixels). Each trial also contained 4 wolf objects (70 × 94 pixels) that had to be avoided or ignored, depending on the predation group of the participant. Target and distractor items (20 pixels) were randomly distributed on a trial-by-trial basis within a regular 10 × 8 virtual grid. During Feature foraging, the 40 targets were yellow and blue disks and the 40 distractors were red and green disks. During Conjunction foraging the 40 targets were red disks and green squares and the 40 distractors were green disks and red squares. In our previous work, we have found no effects of counter-balancing stimulus categories, and used a fixed mapping in the current task to simplify the online protocols.

#### Wolf behaviour

At the start of each trial, the 4 wolf objects were positioned as seen in Fig. 1, at the corners of the dot grid. They immediately began to move, initially converging on the centre of the screen. For wolves that were programmed to move on independent linear trajectories, a new direction was repeatedly chosen from the full 360° range after a period of between 1.7 and 3.3 s. These general motion characteristics were modelled on previous dynamic tasks from our group (e.g., Thornton et al., 2014, 2019) where further methodological details can be found. The lone wolf, if present, changed direction at 20 Hz to converge on the current location of the sheep object. For all wolves, if they arrived at the edge of the dot grid, their direction reversed. Wolf objects did not bounce when colliding with each other, but simply passed through. In the hunted condition, if a wolf overlapped with the sheep object, this terminated the trial. Across trials, the velocity of the 4 wolf objects was either 30, 42, 54, 66 or 78 pixels/s, with 3 repetitions of each velocity randomly distributed across the 15 trials of each condition.

#### Design

Overall the study involved a 2 (Predation: Hunted/Distracted) × 2 (Wolf Behaviour: Pack/Lone) × 2 (Target: Feature/Conjunction) × 5 (Wolf Velocity) factorial design, with the first two factors between subjects and the second two as within subject factors.

#### Task

On each trial, the goal was to cancel all of the target items as quickly as possible by placing the sheep on top of them using the mouse, and then pressing the spacebar. Once selected in this way, items disappeared from the screen. If a distractor item was mistakenly selected, the trial ended. Participants in the hunted condition were required to avoid contact with any of the wolves. For hunted participants, if the sheep object overlapped with any of the wolves, the trial would also end. For participants in the distracted condition, wolf objects could be ignored. A trial would be successfully completed after all 40 targets were cancelled. The game was thus an exhaustive search task, with no opportunity to leave a trial when target prevalence reduced. At the end of each trial an appropriate success or error feedback message was displayed, and the next trial was initiated by pressing a “continue” button. To complete a block of each experimental condition, 15 correct trials were required.

#### Procedure

Participants self-selected the experiment via their https://prolific.co account, and were then directed to the URL of the experiment starting page at https://maltacogsci.org. Here they were shown an introductory screen containing the name of the experiment and identifying the Department of Cognitive Science, University of Malta, as the institution conducting the study. To proceed, participants were asked to navigate to the next page which contained a detailed information and consent form. In order to proceed to the experiment itself, they were required to explicitly confirm their consent. A final screen then provided a reminder of the instructions and that 15 trials of the first condition would follow. After 15 successful trials, a new instruction screen provided details of the target mapping for the conjunction condition. Participants needed to complete 15 of those trials in order to finish the experiment. Block order was fixed, as this factor had not been found to qualitatively affect the pattern of foraging results in our previous work (see Thornton et al., 2019 for a detailed discussion) and in an online context, having the less demanding task first was useful from a familiarisation standpoint.

#### Data analysis

Our primary dependent variable was the average number of runs. As noted above, a “run” corresponds to the sequential selection of targets of the same category. With 40 targets divided into 2 categories, the number of runs on a given trial could vary between 2 and 40. We also examined other dependent variables which have proven sensitive measures of foraging behaviour. These included inter-target times (the time elapsed in milliseconds between two successive target selections) and inter-target distances (the distance in pixels between two successive target selections). On each trial, we also assessed the distance between the sheep object and the closest wolf. This latter measure—Wolf Distance—can provide an indication of whether hunted participants are risk taking or risk averse, with respect to the predator objects. Lastly, search organization was assessed by calculating the “best-*r*” (Woods et al., 2013) that assesses the degree to which target selections were pursued orthogonally (either horizontally or vertically). We calculated the correlation coefficient r1 between the *x* coordinates of all targets in a trial with the order in which they were selected, and the correlation coefficient *r*2 between the *y* coordinates of all targets in a trial with the order in which they were selected. The best-*r* corresponds to the higher of these two correlation coefficients.

All dependent variables were analysed using the same 2 (Predation: Hunted/Distracted) × 2 (Wolf Behaviour: Pack/Lone) × 2 (Target: Feature/Conjunction) × 5 (Wolf Velocity) mixed ANOVA with the first two factors as between subjects and the second two as within subjects, repeated measures. Full details of all analyses can be found in the Open Science Framework (OSF) supplementary material associated with this paper at https://osf.io/jwn8f/, with the text reporting the main factors of interest.

### Results

**Fig. 2:**
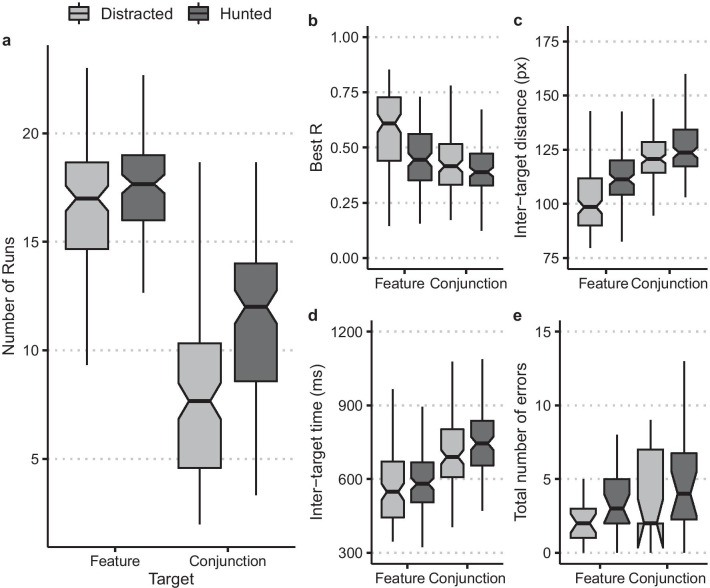
The interaction between Predation (Distracted/Hunted) and Target (Feature/Conjunction) in Experiment 1. Horizontal lines show the central tendency (median) of each distribution, with notches indicating 95% confindence intervals. Boxes identify the upper (75%) and lower (25%) quartiles, with the whiskers indicating maximum and minimum values

Figure 2 summarises the main findings in terms of the interaction between Predation (Distracted/Hunted) and Target (Feature/Conjunction) for each of the dependent variables. Panel a shows that when target identification was easy (Feature condition), both groups of participants switched frequently between target categories, with the number of runs approaching half the total targets (i.e., 20), indicating random selection. Increasing the difficulty of target selection (Conjunction condition) led to a general drop in the number of runs, giving rise to a main effect of Target, *F*(1,44) = 235.5, *p* < 0.001, *η*_*p*_^2^ = 0.58. Of most interest however, is the nature of the Predation × Target interaction, *F*(1, 44) = 7.18, *p* = 0.01, *η*_*p*_^2^ = 0.04. Specifically, the reduction in the number of runs when target selection becomes more difficult is more pronounced for the distracted participants than the hunted participants, the opposite of the pattern we had predicted. Aside from the simple main effect of Predation, *F*(1,44) = 10.7, *p* = 0.002, *η*_*p*_^2^ = 0.09, there were no other significant effects in the analysis of run patterns (see OSF supplementary materials for full descriptive statistics and ANOVA details).

Turning to the additional dependent measures, the only other Predation × Target interaction occurred for best-*r*, *F*(1, 44) = 5.7, *p* = 0.021, *η*_*p*_^2^ = 0.11. As can be seen in Panel b, while search organisation was reduced for both groups of participants during conjunction foraging, distracted participants initially had more regular patterns during the less-demanding feature condition. Panels c–e show the expected main effects of Target for inter-target distances *F*(1,44) = 69.5, *p* < 0.001, *η*_*p*_^2^ = 0.61, inter-target times, *F*(1,44) = 39.9, *p* < 0.001, *η*_*p*_^2^ = 0.48 and number of selection errors, *F*(1,44) = 12.1, *p* = 0.001, *η*_*p*_^2^ = 0.03. That is, participants moved greater distances, selected more slowly and made more selection errors during conjunction than feature foraging.

However, in terms of predation, these measures only gave rise to two significant effects. First, as shown in Panel c, hunted participants generally moved greater distances between selections than distracted participants, giving rise to a main effect of Predation for inter-target distance, *F*(1,44) = 5.4, *p* = 0.025, *η*_*p*_^2^ = 0.11. Second, there was a significant Predation × Wolf-behaviour (Pack/Lone) interaction for inter-target times, *F*(1, 44) = 6.2, *p* < 0.017, *η*_*p*_^2^ = 0.12. While full details of this pattern are given in the OSF supplementary materials, we note that the effect appears to be driven by the distraction condition, where selection speed was significantly slower in the lone wolf condition than the pack condition, (*p* < 0.05, Tukey HSD). Such slowing likely arises when the “ignored” lone wolf approaches the sheep and occludes possible target items. In contrast, the rate of target responses increased slightly for hunted participants in the lone wolf condition, although post-hoc comparisons with the pack condition were not significant, (*p* = 0.92, Tukey HSD).

**Fig. 3:**
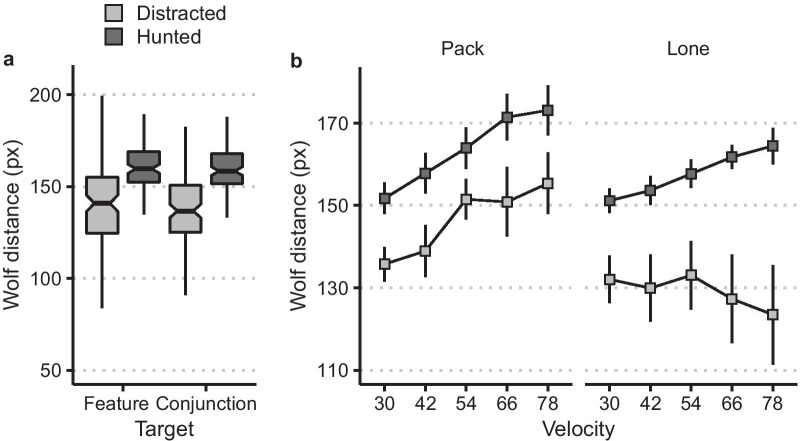
**a** The interaction between Predation (Distracted/Hunted) and Target (Feature/Conjunction) for Wolf Distance. Horizontal lines show the central tendency (median) of each distribution, with notches indicating 95% confindence intervals. Boxes identify the upper (75%) and lower (25%) quartiles, with the whiskers indicating maximum and minimum values. **b** The three-way interaction between Predation (Distracted/Hunted), Wolf Behaviour (Pack/Lone) and Velocity. Error bars indicate 95% confidence intervals

Figure 3 confirms that participants were taking action to avoid being eaten, with the distance to the nearest wolf object at the time of selection, being consistently greater for hunted than for distracted participants, giving rise to a main effect of Predation on Wolf Distance, *F*(1, 44) = 38.1, *p* < 0.001, *η*_*p*_^2^ = 0.35. Remaining with this dependent measure, while neither the Wolf behaviour (Pack/Lone) nor the Wolf Velocity manipulations showed any predation-foraging patterns with respect to the number of runs, there was a clear impact on Wolf Distance. Specifically, there were significant main effects and two-way interactions (see OSF supplementary material) which were in turn qualified by the Predation × Wolf Behaviour × Wolf Velocity interaction, *F*(4, 176) = 4.1, *p* < 0.01, *η*_*p*_^2^ = 0.01, shown in Panel b of Fig. 3. The fairly linear increase in distance seen for both the distracted and hunted groups during the pack condition could be an artefact, reflecting the greater distance travelled by the higher speed wolves. However, during the lone wolf condition, the two lines diverge. For hunted participants, Wolf Distance continues to linearly increase, indicating attempts to avoid being eaten. For distracted participants, the opposite pattern occurs as the “ignored” lone wolf converges on the sheep, and does so more effectively at higher speeds. This pattern provides direct evidence that hunted participants were taking active measures to increase the gap between themselves and the wolves.

**Fig. 4:**
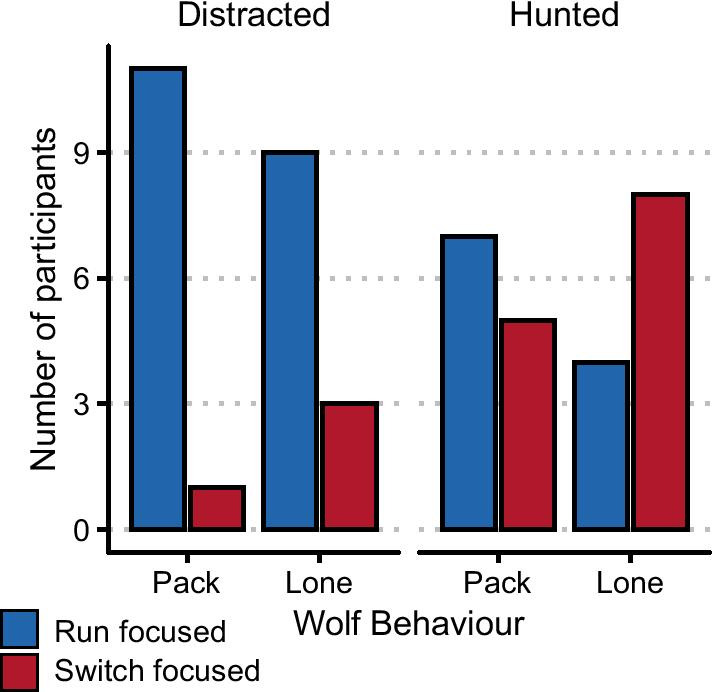
Frequency of foraging strategy as a function of Predation group (Distracted/Hunted) and Wolf Behaviour (Pack/Lone) in Experiment 1. See text for details

Returning to the run patterns shown in Fig. 2a, it is clear that there is considerable variation in performance, particularly in the conjunction condition. A consistent finding in many previous studies from our group, and other labs, has been the existence of subsets of individuals who continue to forage randomly under conjunction conditions (e.g., Clarke et al., 2018; Jóhannesson et al., 2017; Kristjánsson et al., 2014; Tagu & Kristjánsson, 2020; Thornton et al., 2020) The foraging patterns of such individuals is clear to see in the raw data, by plotting the run length for each trial as a function of condition (see Kristjánsson et al., 2014; Fig. 4). Here we provide the equivalent individual plots in OSF supplementary material. As a more concise summary, however, Fig. 4 shows how our 48 participants would be categorised according to whether more than 50% of their conjunction trials are random (switch focused) or non-random (run focused) using a Bonferroni-corrected one-sample runs test (for more details, see Kristjánsson et al., 2019). It is immediately clear that Predation has a large impact on such categorisation, with switch focused foraging being much more prevalent for hunted than the distracted participants. In the General Discussion, we further discuss the possible causes and consequences of such individual foraging behaviour.

Finally, while our main analysis has focused on between-group comparisons, it is also useful to look specifically within the hunted participants. We performed a median split based on the overall number of times participants were eaten by the wolves, to produce a low-surviving “food-focused” group (*M*_eaten events = 13.9, SD = 5.8) and a more successful “wolf-focused” group (*M*_eaten events = 4.7, SD = 1.6), Welch *t*(14.1) = 5.4, *p* < 0.001. We examined performance across the same dependent variables used in the main analysis to explore whether success in avoiding the wolves related to other aspects of foraging behaviour, but there were no clear interactions with this survival variable (see OSF supplementary material for full details). We note that as the “food-focused” individuals would have initiated many more trials than the “wolf-focused” group—trials were terminated with each collision—this additional time and effort does not appear to have affected run behaviour. This is important as it suggests that overall time-on-task—which would have been considerably longer for hunted than distracted participants—is unlikely to affect foraging patterns.

### Discussion

Using an online protocol, we replicated our previous findings that increasing attentional demands using a feature/conjunction manipulation leads to less random foraging behaviour (e.g., Kristjánsson et al., 2014; Tagu & Kristjánsson, 2020; Thornton et al., 2019, 2020). The primary goal of this study, however, was to examine whether foraging patterns changed when participants also had to monitor and avoid potential predators. While our simulated risk of predation manipulation clearly affected performance, it was not in the way we had predicted. Rather than showing a reduced tendency to switch between target categories—the expected effect of increased attentional load—hunted participants continued to alternate, using more frequent, shorter runs than the distracted participants. How might we explain this finding?

One possibility is that the simulated “risk” of predation in our task modulated levels of alertness/arousal (Kahneman, 1973; Posner & Petersen, 1990; Sturm & Willmes, 2001; Yerkes & Dodson, 1908), counteracting the costs of having to both select targets and monitor for wolves. The presence or approach of the predator objects may have actually improved “attentional control” (Kane & Engle, 2003; Unsworth & Robison, 2017), allowing hunted participants to switch more frequently and more efficiently between complex target categories. The effects of phasic changes in alertness and arousal are central to recent attempts to explain individual differences in human cognitive performance (Esterman & Rothlein, 2019; Petersen et al., 2017; Unsworth & Robison, 2017) and more generally play a role in standard capacity models of attention (e.g., Kahneman, 1973) and other relevant models of behaviour (e.g., Aston-Jones & Cohen, 2005; Gray, 1990). The specific suggestion here—which we return to in the General Discussion—is that within-trial modulation in levels of arousal/alertness could have a direct impact on the creation, maintenance and selection of WM search templates during foraging.

Two more directly testable alternative explanations also suggest themselves. First, if attention has to be switched back and forth between target selection and wolf monitoring—as suggested by the MOT/Search study of Alvarez et al. (2005)—then maintaining the focus on a single target category may become more difficult or even impossible, raising the likelihood of a switch. Second, being forced to quickly move from one area of the display to another due to the approach of a dangerous wolf, could simply increase the salience of target items in the proximity of the new landing site, overcoming any tendency to use extended run behaviour. In Experiment 2, we designed a task variant that should help to distinguish between these attention switching and avoidance explanations.

Before leaving Experiment 1, however, we should comment on two other aspects of the results. First, in contrast to our original iPad studies (e.g., Jóhannesson et al., 2017; Kristjánsson et al., 2014), we found little evidence of fully exhaustive category selection during Conjunction foraging in the online task, even for participants in the distracted group. That is, while run length clearly increased when target selection was more demanding, few of our distracted participants consistently selected all of one target category before proceeding to the next (see OSF supplementary figures). We note that in a previous study that used a very similar display and response methodology (Thornton et al., 2019) we also found reduced use of exhaustive runs, which we suggested was an indirect consequence of extended inter-target response times. Specifically, when foraging tempo is quite slow—in the current Experiment 1 average inter-target times are all > 600 ms (Fig. 2)—we would thus expect to see a reduced tendency to use extended runs (see also Thornton et al., 2020 for further discussion).

Second, our attempts to modulate risk by increasing Wolf Velocity or changing Wolf Behaviour were largely unsuccessful, at least in terms of their impact on run patterns. Hunted participants did systematically adjust their distance from the nearest wolf as a function of Wolf Velocity and Wolf Behaviour (Fig. 3), but this did not impact run behaviour. In the General Discussion we suggest some additional ways in which the predictability and/or behaviour of predator objects could be modified in order to increase perceived risk.

## Experiment 2: Sheltering in place

As noted in the Introduction, our primary concern in this paper is to determine whether having to divert attention away from selecting targets affects run patterns in human foraging. While the results of Experiment 1 show that run patterns change between hunted and distracted conditions, there was no evidence that dividing attention led to a reduction in category switches, as we had predicted. To exclude the possibility that spatial avoidance behaviour is somehow masking the tendency to use longer runs, we designed a new task variant where such behaviour was not required. In this way we can also separate the effects of switching attention from the effects of having to physically shift position as the cause of the patterns seen in Experiment 1.

**Fig. 5:**
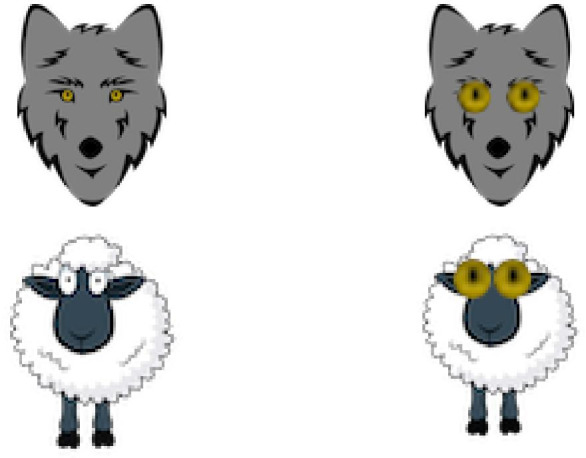
Examples of object changes used in Experiment 2 to signal a pause in feeding, either due to “danger” for hunted participants (Wolf object) or over-feeding for distracted participants (Sheep object)

In the task used in Experiment 2 then, participants had to “freeze” in place in response to danger, rather than avoiding physical contact with the wolves. The same four wolf objects used in Experiment 1 continued to roam the display area during foraging. However, rather than becoming dangerous by physically colliding with the sheep object, we introduced temporal periods when feeding in their presence had dire consequences. Specifically, if the eyes of any of the wolf objects suddenly enlarged, this signalled that they were hungry, and any attempt to collect targets during such a danger period would immediately stop the trial. Figure 5 shows examples of the eye-change events used in Experiment 2, but as before, the task can be played directly online at https://maltacogsci.org/thePredationGame.

The eye-change danger events were randomly distributed in time and occurred unpredictably on only one of the four wolves at any moment. For “hunted” participants, then, successful foraging required frequent checks on the status of the wolf pack in order to avoid being eaten. The physical change from normal to big eyes was designed to be small enough so that it could not be reliably detected peripherally. Danger periods lasted between 1.5 and 3.0 s, and feeding could commence again as soon as the hungry wolf had normal eyes. Foraging for hunted participants was thus divided into “temporal patches” separated by periods of danger. During the danger periods, the movement of the sheep was not constrained, but any attempt to collect targets terminated the trial.

We included two separate groups of control participants. First, to mimic the temporal profile of the hunted group, distracted participants were required to stop feeding periodically, but were not placed in danger of the trial being terminated. This was achieved by having the eyes of the sheep object change from normal to big with the exact same temporal parameters used to control the wolf eyes in the hunted group. The cover story used here was that the sheep would periodically become too sated to eat, and that selection of items was not permitted until the eyes returned to normal. Attempts to cancel items were simply ignored while the sheep had big eyes. While distracted participants thus had to monitor for changes to the display, these always occurred close to the focus of attention (i.e. to the cursor they were using to select), and this group of participants were clearly not required to divert attention to other parts of the display.

The second group of control participants—the baseline group—did not have to alter their foraging behaviour in any way in response to eye changes in the display. Half of the baseline group saw displays in which one of the wolf objects periodically changed, the other half saw displays in which the sheep eyes changed. They were informed that these modifications were designed to distract them, and that they should be ignored. Thus, for all participants in the baseline group, foraging occurred in one continuous temporal period.

If the foraging patterns of hunted participants still resemble those of Experiment 1—with an increase not a decrease in the number of conjunction runs—this would suggest that having to switch attention between monitoring the wolves and foraging is the important factor. If, however, such a tendency is absent or even if we now see the emergence of extended runs, this would indicate that taking avoiding action is the critical factor.

### Methods

#### Participants

A total of 72 new participants were recruited online from https://prolific.co with the group sample size of 3 × 24 designed to match that of Experiment 1. The selection criteria, basic demographics and payment arrangement were the same as in Experiment 1. Here, the mean age was 24.4 years, (SD = 5.5), and there were 28 females. The same Ethics, Data Protection and online protocols were applied as in Experiment 1.

#### Equipment and stimuli

These were the same as Experiment 1, except for the following: The 4 wolf objects now moved by default at the highest velocity used in Experiment 1 (78 pixels/s) and there was no lone wolf. We removed the Velocity and Wolf Behaviour factors from the design as neither had influenced foraging in Experiment 1. Furthermore, in Experiment 2, all of the wolves were programmed to actively avoid the location of the sheep, changing direction and briefly accelerating if they approached within 100 pixels. If there was any overlap between the sheep and wolf, this had no consequences, with the items simply passing through each other. In addition to bouncing off the edges of the screen, the wolf objects now also bounced off each other. These changes were made to maximise the spread of the wolf objects across the display, making the task of monitoring the status of their eyes more demanding. Following the first selection event, a random countdown process was initiated which could last between 1.5 and 5.0 s. When terminated, an eye-change event occurred which lasted between 1.5 and 3.0 s. At the end of the big-eye period, the next change countdown was initiated. The trial was thus divided into a series of normal and big eye periods, the significance of which varied depending on experimental group, as described shortly.

#### Design

Experiment 2 used a 3 (Predation: Baseline/Distracted/Hunted) × 2 (Target: Feature/Conjunction) factorial design, with the first factor between subjects and the second factor within subjects.

#### Task and procedure

The main difference relative to Experiment 1 was that the wolves no longer had to be avoided. The target and distractor items remained the same, as did the requirement to cancel all 40 targets to complete a trial. Hunted participants were required to monitor the eyes of all wolf objects and to refrain from selecting items whenever one of them had big eyes. If a selection was made during a dangerous big eye period, the trial was terminated and a “You’ve been eaten” feedback message displayed. To account for movements that may have been initiated as an eye change event occurred, a grace period of 500 ms was applied after the onset of the danger event.

For distracted participants, selection was disabled whenever the eyes of the sheep enlarged. They were thus required to pause with the same temporal frequency as the hunted participants. Baseline participants were equally divided into wolf eyes and sheep eyes conditions, but were told to ignore the changes and were able to forage continuously throughout the trial. All participants completed 10 correct feature trials and 10 correct conjunction trials. The number of trials was reduced relative to Experiment 1, to compensate for the longer trial duration caused by the interleaved big-eyes periods.

#### Data analysis

We used the same set of dependent variables as in Experiment 1 which were analysed using a 3 (Predation: Baseline/Distracted/Hunted) × 2 (Target: Feature/Conjunction) mixed ANOVA. Full tables and analysis results are provided in the OSF supplementary materials.

### Results

**Fig. 6:**
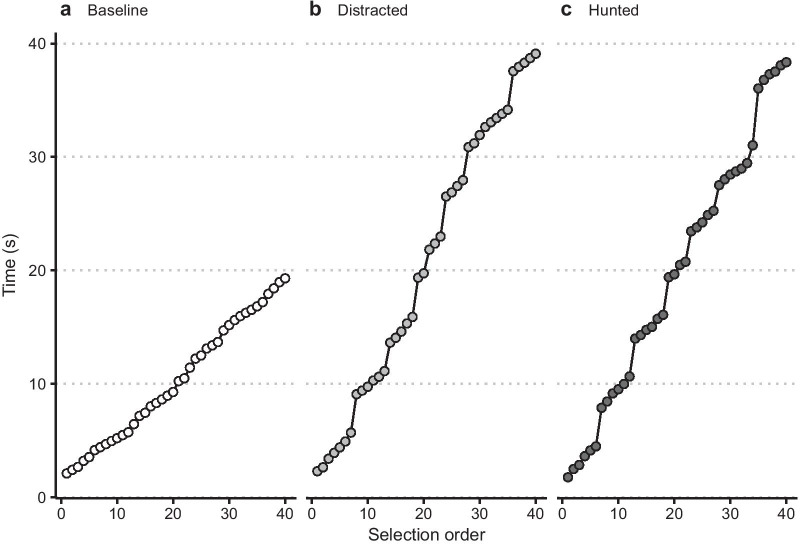
The time course of three example trials, illustrating how eye-change periods affected the structure of foraging for each group of participants. The panels show a single trial from the Conjunction condition of one participant. Each dot represents a collection event, and target type is not indicated. See text for further details

Figure 6 confirms how the temporal profile of trials varied across Predation condition. For baseline participants, foraging proceeded in a single episode. For both distracted and hunted participants, however, the eye-change events required pauses in target selection, leading to distinct “temporal patches” and an overall increase in trial duration.

**Fig. 7:**
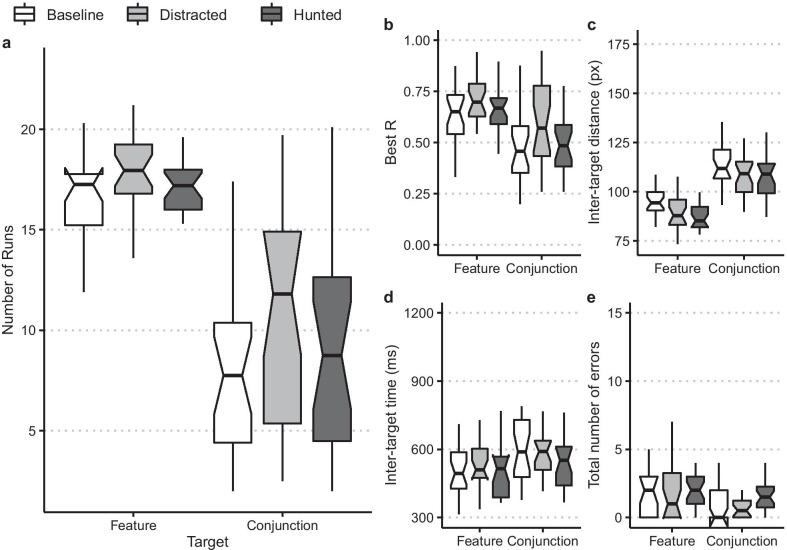
The interaction between Predation (Distracted/Hunted) and Target (Feature/Conjunction) in Experiment 2. Horizontal lines show the central tendency (median) of each distribution, with notches indicating 95% confindence intervals. Boxes identify the upper (75%) and lower (25%) quartiles, with the whiskers indicating maximum and minimum values

Figure 7 summarizes the main findings of Experiment 2 in terms of the interaction between Predation (Baseline/Distracted/Hunted) and Target (Feature/Conjunction) for each of the dependent variables. For the number of runs, there continued to be a clear impact of Target, with random selection during Feature foraging but significantly fewer runs during Conjunction foraging, *F*(1,69) = 163.2, *p* < 0.001, *η*_*p*_^2^ = 0.49. In terms of Predation, and in contrast to Experiment 1, distracted participants now had a slightly higher tendency to switch during conjunction trials, although neither the main effect of Predation, *F*(2,69) = 2.7, *p* = 0.08, *η*_*p*_^2^ = 0.04, nor the Predation × Target interaction, *F*(2,69) = 0.9, *p* = 0.42, *η*_*p*_^2^ = 0.01, were significant. More generally, there was still no evidence that having to divide attention increased the use of extended run events relative to baseline, even in the absence of avoidance behaviour.

Analysis of the other dependent variables (Fig. 7, Panels b–e) largely mirrored this pattern, with significant effects of Target, but no influence of Predation (see OSF supplementary material). The only exception to this general pattern was for inter-target distance, where there was a main effect of Predation, *F*(2,69) = 4.2, *p* = 0.02, *η*_*p*_^2^ = 0.08. As can be seen in Panel c, the two groups that foraged in discrete periods moved shorter distances than the baseline participants, with Tukey post-hoc tests showing this difference to be significant for the hunted group, *t*(69) = 2.75, *p* = 0.02 and marginal for the distracted group, *t*(69) = 2.4, *p* = 0.07. The difference between the hunted and distracted group was not significant, *t*(69) = 0.4, *p* = 0.9. Although not shown, we also note that, as intended, Wolf Distance no longer varied across any of the factors, confirming that participants did not attempt to avoid the predator objects.

**Fig. 8:**
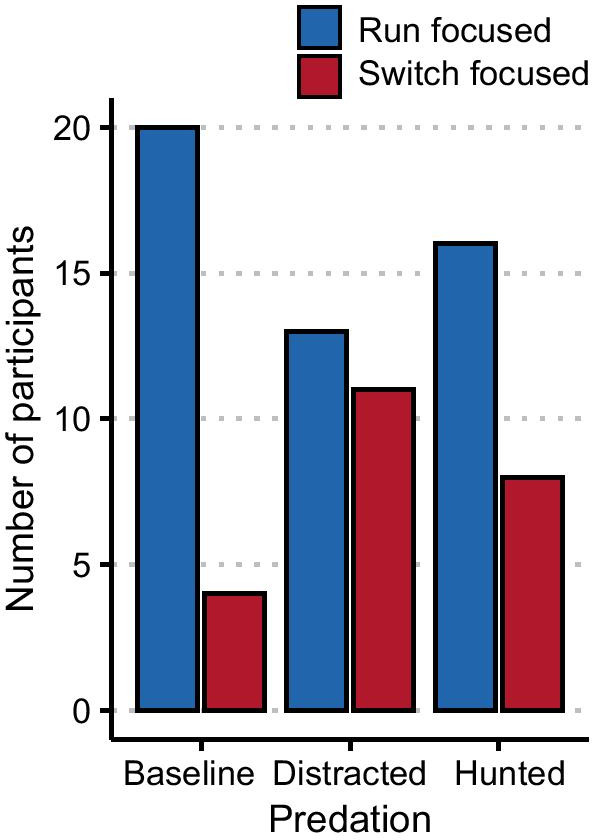
Frequency of foraging strategy as a function of Predation group in Experiment 2. See text for details

Following the analysis in Experiment 1, Fig. 8 shows how participants are classified in terms of their use of random selection during conjunction trials (see OSF supplementary material for individual participant plots). Compared to baseline, both distracted and hunted groups contain more switch-focused individuals who consistently used non-random switching during conjunction trials, patterns that are consistent with the run data shown in Fig. 7a. Again, this increased use of switching in the presence of any form of secondary task argues against a simple model where increases in attentional load necessarily lead to the use of extended runs.

**Fig. 9:**
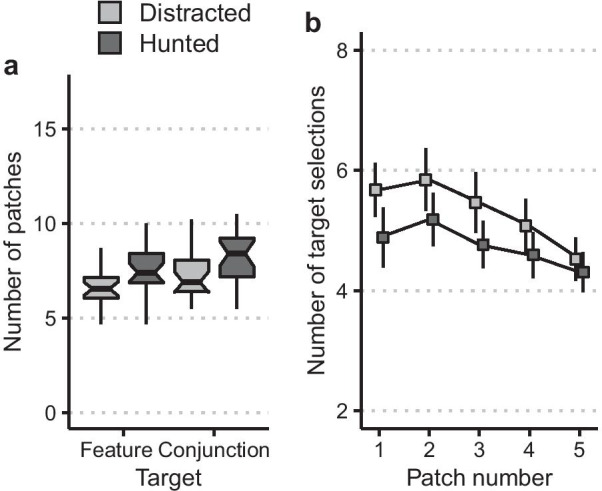
**a** Number of patches as a function of Predation (Distracted/Hunted) and Target (Feature/Conjunction). Horizontal lines show the central tendency (median) of each distribution, with notches indicating 95% confindence intervals. Boxes identify the upper (75%) and lower (25%) quartiles, with the whiskers indicating maximum and minimum values. **b** Average number of targets per patch as a function of Predation. Error bars indicate 95% confidence intervals

For the Distracted and Hunted groups, we also looked at performance within and across the “temporal patches” caused by the appearance of wolf or sheep eye changes. Panel a of Fig. 9 shows how the number of patches varied across conditions, with the only significant effect being a main effect of Target, *F*(1,46) = 19.2, *p* = 0.01, *η*_*p*_^2^ = 0.01. The average number of target items per patch did show some modulation as a function of group. Specifically, there was a Predation × Patch Number interaction, illustrated in Panel b of Fig. 9. The Hunted participants appeared to select slightly fewer targets during the early part of the trial. Note that this figure collapses across Target, and we also restricted analysis to the first 5 patches, as the number of samples at later stages in the trial was too variable. There were no clear patterns in terms of number of runs per patch or any of the other dependent measures (see OSF supplementary material for full analysis).

Finally, for the hunted group, we categorised participants in terms of those who consistently continued to feed during danger periods from those that did not. The “food-focused” group (*M*_eaten events = 15.2, SD = 5.6) were eaten consistently more often—so more trials had to be replaced—than the “wolf-focused” group (*M*_eaten events = 5.4, SD = 2.7), Welch *t*(15.9) = 5.4, *p* < 0.001. Nevertheless, as in Experiment 1, this analysis did not show any changes in foraging behaviour as a function of focus group (see OSF supplementary materials).

### Discussion

There were two main findings from Experiment 2. First, the tendency for hunted participants to switch more frequently than distracted participants was not replicated. This clearly suggests that the pattern observed in Experiment 1 was related to avoidance behaviour, rather than the demands of switching between the tasks of monitoring the wolves and foraging. Second, with avoidance behaviour removed from the task, we found no evidence that having to attend to other aspects of the display—either the distributed wolf objects or the eyes of the more central sheep—resulted in an increase in extended runs relative to baseline. Rather, here, there was a hint that the Distraction condition promoted the tendency to switch more frequently during conjunction conditions than the baseline participants, both in terms of the number of runs and the categorisation of individual participants. At least in this scenario, then, dividing attention does not appear to lead to extended run behaviour as we had predicted. We return to this issue in the General Discussion.

## Experiment 3: Risk versus reward

Experiments 1 & 2 suggest that spatial disruption caused by the need to physically avoid predator objects is a more reliable modulator of run behaviour in our tasks than the need to allocate or switch attention between tasks. In Experiment 3, we asked whether the need to avoid predators would also interact with the ability to strategically prioritise high-reward targets. Foraging behaviour in the wild is thought to reflect a delicate balance between risk and reward (Gilliam & Fraser, 1987; Houston et al., 1993; Kotler & Brown, 2017; Lima & Dill, 1990). Here, a new group of 48 participants played a version of our task in which we simultaneously reduced the prevalence and increased the value of one of the target categories (Wolfe et al., 2018).

Rather than exhaustively searching for all possible targets, here in order to complete a trial, participants needed to score 30 points by selecting a subset of the 40 available targets. On every trial, there were 10 yellow targets, each worth 2 points, and 30 blue targets, each worth 1 point, randomly distributed amongst 40 distractors (20 red and 20 green items). The optimal strategy would be to prioritise selection of the high reward yellow targets as this would require fewer collection episodes. As in Experiment 1, half of the participants were hunted by the wolves, and half could ignore them. In addition to our standard foraging measures, we also computed a “selection order” score for both high and low reward target categories, where a lower score indicates earlier selection during a trial. For distracted participants, we predicted that high reward targets should be preferentially selected, leading to lower selection order scores than for low reward targets. Our primary question was whether hunted participants would also show such a pattern, or whether the risk of predation would disrupt such an optimal strategy. Note that we did not include a feature/conjunction manipulation in this task, as we wanted baseline run patterns to be determined by target value rather than selection difficulty. As conjunction foraging would be expected to induce the use of long runs, we chose to use a feature task, where only expected reward should promote such behaviour.

### Methods

#### Participants

A new group of 48 participants were recruited online from https://prolific.co. The selection criteria, basic demographics and payment arrangement were the same as in Experiments 1 & 2. Here, the mean age was 24.9 years, (SD = 6.0), and there were 20 females. The sample size was also determined and verified as in Experiments 1 & 2, and the same Ethics, Data Protection and online protocols were applied.

#### Equipment and stimuli

These were the same as Experiment 1, except for the following: on each trial, the 40 target items now always consisted of 10 high-value yellow disks (2 points) and 30 low-value blue disks (1 point). The distractors were red and green disks. We did not use a Feature/Conjunction manipulation in this experiment. The 4 wolf objects now always moved at the highest velocity used in Experiment 1 (78 pixels/s) and there was always a lone wolf that directly tracked the position of the sheep object.

#### Design

Experiment 2 had a simplified 2 (Predation: Hunted/Distracted) × 2 (Reward: High/Low) factorial design, with the first factor between subjects and the second factor within subjects.

#### Task and procedure

The only difference relative to Experiment 1 was that a trial now terminated when the cumulative points score reached 30. While the values of the two types of target were pointed out, no further guidance or suggestions on search strategy were provided. All participants completed 20 correct trials with the same target/distractor mappings.

#### Data analysis

For the sake of completeness, we conducted a full analysis of foraging behaviour using the same set of dependent variables as in Experiments 1 & 2. However, some caution is needed in interpreting such analysis as with a high-value versus low-value design the target categories were not equally prevalent on the screen as they were during both feature and conjunction foraging in previous tasks. As high-value targets are less numerous than low-value targets, runs will be mechanically shorter, and targets are more distributed on the screen, leading to higher inter-target distances and inter-target times, for example. Nevertheless, we present the full analysis in the OSF supplementary material and note here that the general pattern of Predation main effects were consistent with those seen in Experiment 1.

The main dependent measure of interest in the current task is “Selection Order”, which is used to quantify at which stage during a trial the high or low value targets are selected. Essentially, each item is simply weighted by its serial position during the trial, with lower values indicating earlier selection. We analysed the Selection Order patterns using a 2 (Predation: Hunted/Distracted) × 2 (Reward: High/Low) mixed ANOVA. Full descriptive statistics and analysis details are provided in the OSF supplementary materials.

### Results

**Fig. 10:**
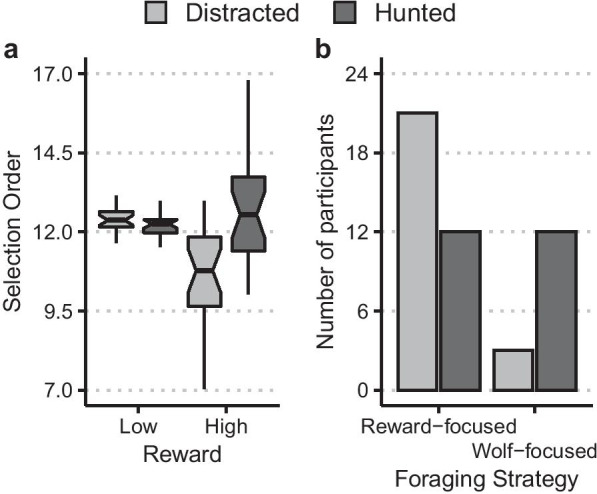
**a** Selection Order as a function of Predation (Distracted/Hunted) and Reward (High/Low). Horizontal lines show the central tendency (median) of each distribution, with notches indicating 95% confindence intervals. Boxes identify the upper (75%) and lower (25%) quartiles, with the whiskers indicating maximum and minimum values. **b** Frequency of foraging strategy as a function of Predation group in Experiment 3. See text for details

Figure 10a shows Selection Order as a function of Predation and Target Value. The analysis of these patterns revealed a clear interaction between these factors, *F*(1, 46) = 15.9, *p* < 0.001, *η*_*p*_^2^ = 0.26. Distracted participants clearly prioritised the high value targets, seeking them out and selecting them earlier during a trial than the low value targets (High value: *M* = 10.7, SD = 1.5; Low value: *M* = 12.5, SD = 0.8; *p* < 0.0003 at Tukey HSD post-hoc tests), a strategy that minimised the number of collection episodes needed to complete the task. Hunted participants, however, showed no overall bias towards the high value targets (High value: *M* = 12.7, SD = 1.8; Low value: *M* = 12.2, SD = 0.4; *p* = 0.63 at Tukey HSD post-hoc tests), indicating that predator behaviour disrupted the optimal strategy.

Interestingly, there were clear individual differences in strategy within the hunted, but not the distracted group. Figure 10b shows a categorisation of participants in terms of whether their individual selection order scores were on average lower for high-value targets than low-value targets (reward focused) or whether this pattern was absent (wolf focused). While the distracted group very consistently show the reward focused pattern, the hunted group are exactly divided between the two strategies.

### Discussion

In Experiment 3, we explored the relationship between risk of predation and reward. As expected, in general, those participants who needed to both monitor and avoid the wolves were less likely to use a strategy that would minimise their selection episodes, by prioritising the high-value targets. We also replicated the basic finding from Experiment 1 that hunted participants favour shorter runs, travel further distances and show less organised search. As might be expected, they also keep a larger distance from the nearest wolf object (see OSF supplementary analysis). Altogether then, the findings demonstrate that simulated risk of predation in our online scenario does affect foraging patterns in the context of target reward.

Perhaps the most interesting aspect of these results, however, is the fact that the hunted participants could be equally divided into two clear categories. The reward-focused participants were able to cope with the presence of the “dangerous” wolves, and still prioritise the high-value targets. Although we can only speculate given the currently available demographics—gender and age were not predictive—it seems possible that reward-focused participants were more confident/experienced at rapidly controlling the sheep cursor in close proximity to the wolves, thus allowing them to use the same strategy employed by distracted participants who could completely ignore the predators. Alternatively, reward-focused participants may simply be less risk averse or more sensation seeking. It would clearly be interesting to further explore these individual differences.

## General discussion

In three experiments, we explored how simulated risk of predation affects patterns of human foraging in an online scenario. In Experiment 1, we used an established Feature/Conjunction manipulation to vary the difficulty of target selection and required “hunted” participants to physically avoid the approach of predator objects. In Experiment 2, we used the same Feature/Conjunction manipulation, but required participants to monitor the wolf objects and “freeze” in place, stopping feeding during periods of danger, rather than moving to avoid them. In Experiment 3, we varied the value and the prevalence of target items to examine potential trade-offs between risk and reward, using the same physical avoidance task used in Experiment 1. In the remainder of the General Discussion, we briefly summarise our main findings, before considering what the current results might tell us about dividing attention during multiple-item search. Finally, we make some suggestions for future research directions with our novel predation scenario and reflect on our experience of collecting this foraging data in an online scenario.

In both Experiments 1 & 2, we were able to replicate previous findings that participants adapt their foraging patterns depending on the attentional effort required to select individual targets (e.g., Kristjánsson et al., 2014; Kristjánsson et al., 2018; Kristjánsson et al., 2020b; Tagu & Kristjánsson, 2020; Thornton et al., 2019, 2020). When targets could be selected based on a single colour feature, participants switched freely between both available categories. When targets were defined in terms of a conjunction of colour and shape, there was an overall shift towards selecting items in extended runs from the same category. In addition to run patterns, we also observed changes in other dependent measures—such as inter-target distance and search organization (best-*r*)—that were also consistent with less random foraging behaviour.

Our goal in these experiments, however, was to establish how such patterns of foraging change when participants were also required to perform a secondary task. Our main prediction—based on general dual-task logic in humans and the known behaviour of animals exposed to risk of predation—was that hunted participants would switch less often between target categories, leading to fewer, longer runs than those patricipants who were not being hunted. Contrary to our prediction, in Experiment 1, hunted participants were actually more likely to switch between target categories than distracted participants. In Experiment 2, we showed that this pattern was probably caused by the need to physically shift position unpredictably within the display in order to avoid the wolves. That is, when the need to take avoiding action was removed, but hunted participants were still required to monitor the status of predator objects, there was no change in run behaviour relative to baseline. In Experiment 3, the disruptive effect of having to physically avoid predator objects was confirmed when we were able to show that this also alters the ability to prioritise high reward items.

Overall, then, we found little evidence that patterns of run behaviour within our multi-target foraging task were disrupted by the need to attend to additional “predator” elements. This confirms findings from the human search literature, reviewed in the Introduction, where performing a secondary task while searching has often been achieved with minimal cost (e.g., Alvarez et al., 2005; Drew et al., 2016; Woodman et al., 2001). In particular, our results from Experiment 2 seem to support the conclusions of Alvarez et al. (2005) who suggested that tracking and search can be interleaved quite successfully by rapid attention switching between the two tasks. Clearly, while the current work was inspired by the idea of predation in the wild, the task demands of our online game would seem to place the scope of our findings firmly within the domain of human search performance rather than animal predation literature. Below, we consider this implication in more detail and speculate on ways in which future studies with our task might more successfully bridge the gap between human and animal foraging.

One further aspect of the current data that deserves to be highlighted was the appearance of clear individual differences in foraging strategy within each of the experiments. In Experiment 1, for example, the additional load of avoiding the wolves increased the prevalence of individuals who continued to switch between target categories during conjunction foraging compared to the distracted participants (Fig. 4). In our earlier work, we had termed individuals who displayed such behaviour “super-foragers” (e.g., Jóhannesson et al., 2017; Kristjánsson et al., 2014)—after the “supertaskers” of Watson and Strayer (2010)—to reflect their apparent resistance to increased cognitive load. In our standard foraging tasks, we typically see about 25% of each sample who spontaneously adopt this strategy. Understanding the factors that influence the choice of run-focused or switch-focused conjunction behaviour is clearly of future interest.

We had initially pursued the idea that the ability to switch easily during conjunction foraging reflected stable individual differences in more general cognitive abilities, such as WM or attention span. However, the story appears to be more complex. For example, while such links between foraging ability and cognitive measures have been found in children (Ólafsdóttir et al., 2016, 2019), this is not the case with adults (Clarke et al., 2020; Jóhannesson et al., 2017). Furthermore, several studies have suggested that the choice to switch categories rather than use extended runs during conjunction foraging may be under more strategic control. In one variant of our original iPad task we manipulated the overall duration of trials (Kristjánsson et al., 2018). When observers had only 5, 10 or 15 s to select targets, they switched far more than when they had unlimited time. We suggested that adding time pressure in this way changed levels of concentration, facilitating switching. Similarly, when we explicitly controlled the tempo at which participants were allowed to respond (Thornton et al., 2020), we found that the majority were able to switch frequently given sufficient time between selection events (see also Prpic et al., 2019; Thornton et al., 2019).

These findings suggest that the use of extended runs in our original iPad tasks occurred, at least in part, because participants chose to prioritize response speed, making switching under conjunction conditions more effortful and thus less appealing (Gray & Boehm-Davis, 2000; Gray & Fu, 2004; Gray et al., 2006). Tagu and Kristjánsson (2020) also recently found that during eye-gaze foraging (Jóhannesson et al., 2016), error rates were lower for those who used extended runs compared to those that would typically be classified as switch-focused, suggesting that the former were simply more averse to making errors. In the current experiments, all groups where the foraging task was modified—either with a danger component (i.e., hunted groups in each experiment) or simply having to pause during foraging (i.e., distracted participants in Experiment 2)—showed an increase in the number of switch-focused participants (Figs. 4, 8, 10) relative to the baseline groups. Again, this argues that task factors must interact with individual preferences or abilities to determine the foraging strategy a given participant will adopt (see Clarke et al., 2020; Nowakowska et al., 2021; Thornton et al., 2020 for related discussion).

In its current form, our online predation game probably has little in common with foraging in the wild. There are several ways in which the task could be adapted so that findings in human participants might have more relevance to the behavioural ecology literature that inspired it. We are grateful to our reviewers for pointing us in several interesting directions. Overall task demands could be increased by making the selection of target items more demanding, either by reducing their visibility, prevalence or identity. Distributing target items in patches—which themselves could vary in likelihood of harbouring or attracting predators—might be another approach. Rather than using an exhaustive design where all target items must be collected, giving participants the ability to leave a patch or a trial when reward levels drop below some threshold may interact in interesting ways with perceived risk. For example, risk of predation may modulate the balance between explorative versus exploitative search strategies (e.g., Cohen et al., 2007; Hills et al., 2015) known to play a role in many different contexts (e.g., Chin et al., 2015; Kane et al., 2017; Wiegand et al., 2019).

Another relatively easy-to-implement approach would be to modulate the predictability of predator appearance. While the task we introduced in Experiment 2 required monitoring of constantly present items which could periodically become dangerous, the eye change events used may well have lacked a sense of danger or urgency. Providing peripheral cues that indicate the probability of an attack, and having predators appear and more actively hunt the sheep object may well increase the sense of risk. As we mentioned in the Discussion of Experiment 1, we believe that exploring how phasic changes in arousal/alertness (Esterman & Rothlein, 2019; Kane & Engle, 2003; Petersen et al., 2017; Unsworth & Robison, 2017) affect target template switching during foraging could be a very useful direction for future research. That is, in addition to known roles in modulating exploratory behaviour in general (e.g., Cohen et al., 2007; Hills et al., 2015) it may be useful to include arousal/alertness as a more explicit factor in models that seek to explain the ability and/or preference to switch between target templates in memory (e.g., Dukas, 2004; Kamil & Bond, 2006; Kristjánsson & Kristjánsson, 2018; Kristjánsson et al., 2014). While we are clearly limited in our ability to modulate arousal/alertness in either lab based or online tasks—relative to an animal being hunted in the wild—exploring other ways to simulate danger and/or increasing the costs associated with failure may well prove fruitful.

Finally, we should note that this was our first experience in designing a task and collecting data specifically for an online setting, an adventure undertaken directly as a consequence of the COVID-19 pandemic that closed many labs during 2020. While it is clear that some compromises have to be made in terms of display environment and task length, we were still easily able to replicate our findings from previous lab studies. We thus echo the more general message from several recent review papers that collecting data online is a real option for vision/attention/cognition researchers (e.g., Bridges et al., 2020; Chetverikov & Upravitelev, 2016; Miller et al., 2018; Pronk et al., 2019). Furthermore, having access to a very large and diverse participant database—particularly ones with high professional and ethical standards such as https://prolific.co—augers well for future studies that want to focus on individual differences, whether in the context of foraging, as here, or beyond.

## Data Availability

Copies of the source code and analysis scripts are available upon request. Raw data files, descriptive statistics and additional data analysis reports have been uploaded as supplementary material to the Open Science Foundation (OSF) page associated with this paper at https://osf.io/jwn8f/.

## Footnotes

1 While our online task was inspired by the animal literature on predation, our goal in the current paper is to better understand human foraging under conditions of divided attention. It is clear that the perceived and actual risks faced by prey in the wild are very different from those experienced by our participants, and thus our findings should be interpreted accordingly.
